# Assessing Clinical Effectiveness of Ceftolozane/Tazobactam, Ceftazidime/Avibactam, and Meropenem/Vaborbactam in Bacteremia: A Systematic Review and Meta-Analysis

**DOI:** 10.3390/antibiotics15090843

**Published:** 2026-08-31

**Authors:** Shahd Mohammad, Mosab Albalas, Mohammad M. Al-Ahmad

**Affiliations:** 1 Clinical Pharmacy Department, Abu Dhabi Health Services Company—SEHA, Abu Dhabi P.O. Box 109090, United Arab Emirates or shahdmufid@gmail.com (S.M.); mbalas@seha.ae (M.A.); 2 Clinical Pharmacy Department, College of Pharmacy, Al Ain University, Abu Dhabi P.O. Box 64141, United Arab Emirates

**Keywords:** bacteremia, Ceftolozane/Tazobactam, Ceftazidime/Avibactam, Meropenem/Vaborbactam, meta-analysis

## Abstract

**Background/Objectives:** Bacteremia poses a significant clinical challenge, often necessitating the off-label use of novel β-lactam/β-lactamase inhibitor (BL/BLI) combinations such as Ceftolozane/Tazobactam (C/T), Ceftazidime/Avibactam (C/A), and Meropenem/Vaborbactam (M/V). Despite their widespread use, no conclusive data support their effectiveness in bacteremia. This meta-analysis aims to evaluate the clinical effectiveness of C/T, C/A, and M/V in treating bacteremia, with a focus on survival outcomes and clinical cure rates. **Methods:** A systematic review and meta-analysis were conducted following a comprehensive search of Scopus and PubMed databases. Studies reporting survival and clinical cure rates in patients treated with C/T, C/A, or M/V for bacteremia were included. Data extraction followed predefined criteria, and a random-effects meta-analysis was performed to synthesize the findings. **Results:** Twenty studies met the inclusion criteria, with 18 reporting survival outcomes and 10 evaluating clinical cure rates. The pooled survival and clinical cure rates were 73.6% (95% CI: 67.5–79.8%, *p* < 0.001) and 76.2% (95% CI: 69.2–83.3%, *p* < 0.001), respectively, both exhibiting moderate heterogeneity. Subgroup analyses revealed consistent survival trends across study quality, with low- and high-risk-of-bias studies demonstrating comparable estimates. Given that C/A was the most frequently studied agent, its dedicated subgroup analysis yielded a pooled survival estimate of 73.4% (95% CI: 66.4–80.3%, *p* < 0.001). **Conclusions:** This meta-analysis provides supporting evidence for the clinical effectiveness of C/T, C/A, and M/V in bacteremia reinforcing the role of BL/BLIs in antimicrobial stewardship as viable options for resistant infections. However, further randomized controlled trials (RCTs) are warranted to validate these results and refine treatment strategies for bacteremia.

## 1. Introduction

Antimicrobial resistance (AMR) has emerged as a critical global health challenge, threatening the efficacy of existing antibiotics and complicating the treatment of bacterial infections [1]. The widespread misuse and overuse of antibiotics in clinical and community settings have accelerated the emergence of multidrug-resistant organisms (MDROs), particularly carbapenem-resistant *Enterobacteriaceae* (CRE) and multidrug-resistant *Pseudomonas aeruginosa* (MDRPA) [2]. These pathogens are associated with substantial morbidity and mortality, further compounded by the limited availability of effective therapeutic options. For bloodstream infections (BSIs), including bacteremia, AMR presents an even greater clinical challenge due to the high risk of progression to sepsis and septic shock, both of which are associated with significant mortality rates and substantial healthcare costs [3].

The development of novel β-lactam/β-lactamase inhibitor (BL/BLI) combinations, including Ceftolozane/Tazobactam (C/T), Ceftazidime/Avibactam (C/A), and Meropenem/Vaborbactam (M/V), represents a major advancement in the treatment of infections caused by resistant Gram-negative bacteria (GNBs). These agents target key resistance mechanisms in GNBs: C/T is effective against MDRPA, C/A primarily addresses CRE, and M/V combines broad-spectrum carbapenem activity with potent inhibition of carbapenemase enzymes [4]. Although these antibiotics are FDA-approved for indications such as complicated urinary tract infections (cUTIs) and complicated intra-abdominal infections (cIAIs), they are increasingly utilized off-label for treating bacteremia.

Bacteremia, defined as the presence of bacteria in the bloodstream, is a severe clinical condition with significant morbidity and mortality. It is estimated to occur in 150 to 300 cases per 100,000 individuals annually, with higher prevalence in hospital settings, particularly among critically ill patients in intensive care units (ICUs). Risk factors for bacteremia include immunosuppression, advanced age, chronic health conditions, and invasive procedures [5]. The increasing prevalence of MDROs in bacteremia further complicates management, as traditional antibiotics often fail to provide adequate coverage. In this context, a growing body of real-world evidence has evaluated the effectiveness of C/T, C/A, and M/V in bacteremia, underscoring their therapeutic value. However, with antimicrobial resistance on the rise, assessing the utility of these agents and ensuring their judicious use is critical to preserving their efficacy. Therefore, this meta-analysis synthesizes all available observational data assessing the off-label use of C/T, C/A, and M/V in bacteremia, enhancing the statistical power of evidence to guide optimal treatment strategies.

This review exclusively includes studies that independently evaluated these antibiotics, without comparator groups, with the primary objective of determining their clinical effectiveness, focusing on survival and clinical cure rates associated with their use in managing bacteremia in adult patients.

## 2. Results

**Figure 1:**
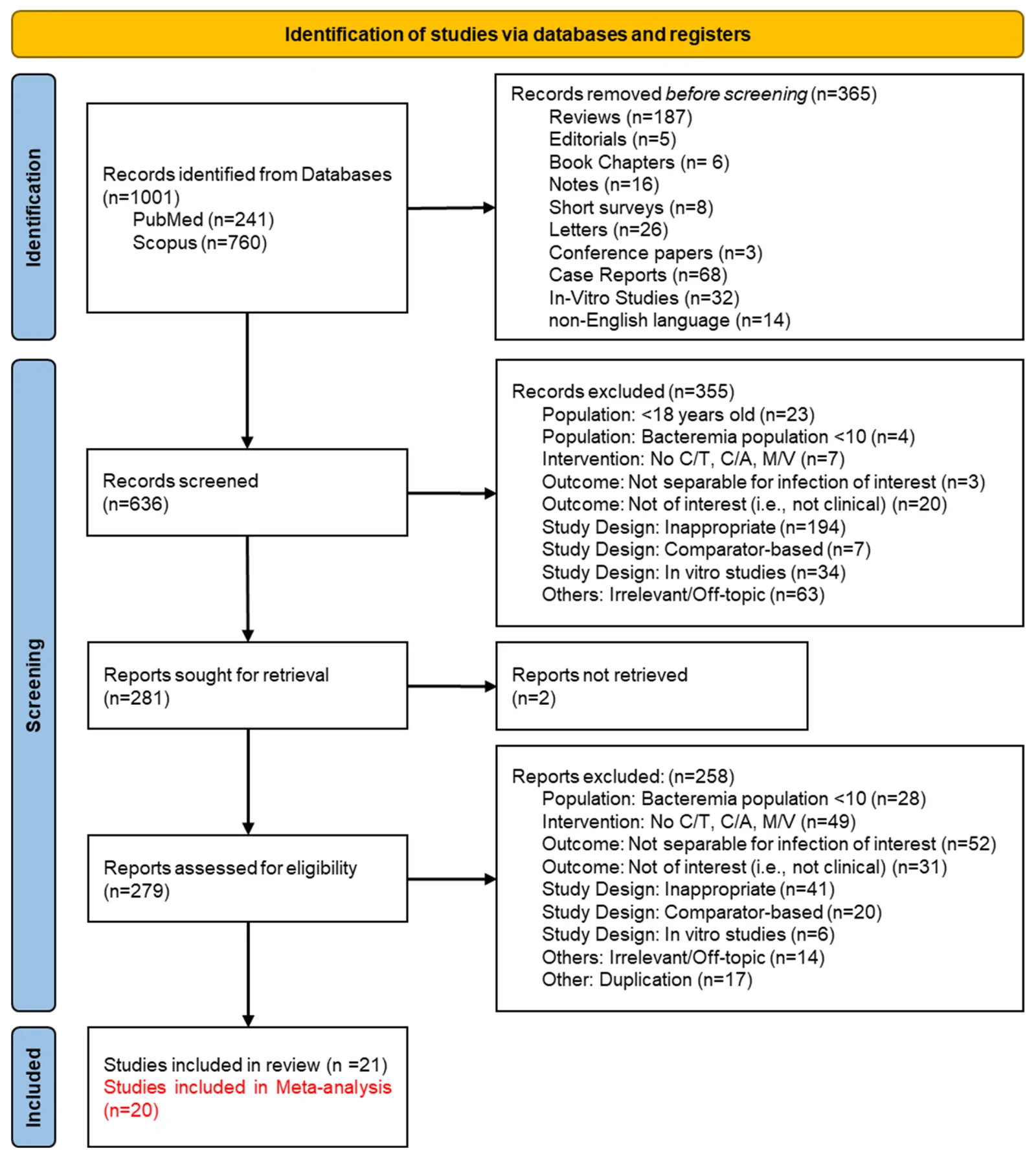
Publication selection flowchart.

A total of 1001 publications were identified through database searches, including 241 from PubMed and 760 from Scopus (Figure 1). After excluding 365 articles based on predefined criteria, 636 titles and abstracts were screened, with 355 articles excluded for irrelevance or failure to meet inclusion criteria. Full-text review was conducted for 281 studies, resulting in the exclusion of 243 articles due to insufficient bacteremia populations (<10 patients), ineligible interventions (no C/T, C/A, or M/V), or outcomes not specific to bacteremia. This left 21 eligible studies, with 20 included in the meta-analysis. One comparative study evaluating C/A vs. M/V was excluded to minimize heterogeneity but was narratively synthesized to enhance supplementary clinical insights and comprehensiveness.

The 20 included studies, published between 2017 and 2024, provide a global perspective on the off-label use of C/T, C/A, and M/V for the treatment of bacteremia. These studies were geographically distributed across the United States (30%) [6,7,8,9,10,11], Italy (30%) [12,13,14,15,16,17], Spain (15%) [18,19,20], China (10%) [21,22], and other regions, including Saudi Arabia, Brazil, and India (15%) [23,24,25]. Study durations varied between 6 and 67 months, with an average of approximately 29 months.

The majority of the included studies (85%) were retrospective observational designs, collectively analyzing 520 bacteremia cases with sample sizes ranging from 10 to 104 patients (Table 1). Key pathogens included CRE, primarily *Klebsiella pneumonia*, and MDRPA, underscoring their critical role in multidrug resistant (MDR) infections. Secondary bacteremia was more frequently reported than primary bacteremia, ranging from 13% to 86.8%, primarily stemming from respiratory, urinary, and intra-abdominal infections. Primary bacteremia was less common, ranging from 10.4% to 33.3%, and rarely reported exclusively (Table 1).

C/A was the most extensively studied antibiotic, featured in 75% of the included studies and predominantly targeting CRE infections. C/T was evaluated in 15% of the studies, while M/V was included in 10% of the studies. Standardized dosing regimens were commonly reported, with adjustments for renal function. C/A was administered as 2.5 g every 8 h, C/T as 1.5–3 g every 8 h depending on severity, and M/V as 4 g every 8 h. Approximately 30% of the studies utilized BL/BLIs in combination with other agents [6,15,17,24], such as aminoglycosides or colistin, to enhance efficacy against severe MDR infections. Notably, one study evaluated C/A in combination with Aztreonam for coverage against New Delhi metallo-beta-lactamase (NDM)-producing organisms, addressing resistance mechanisms not covered by C/A alone [14].

**Figure 2:**
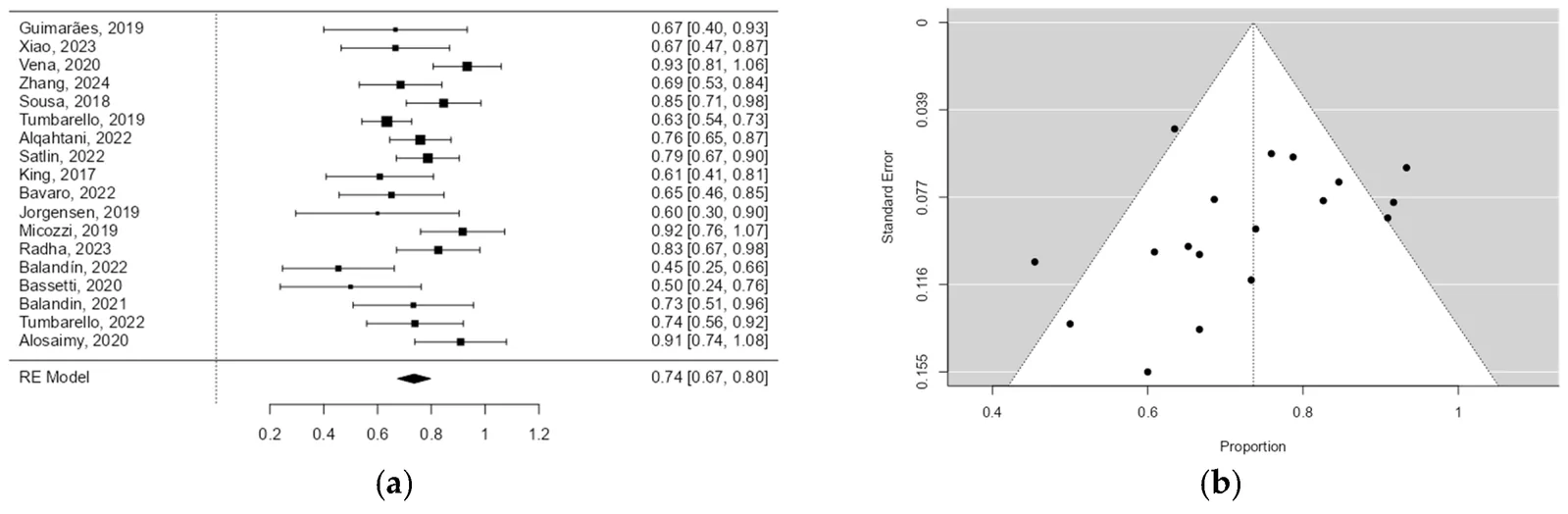
(**a**) Forest plot of pooled survival estimate. (**b**) Funnel plot for assessing publication bias in survival outcome. Symmetry indicates minimal risk of substantial bias.

**Figure 3:**
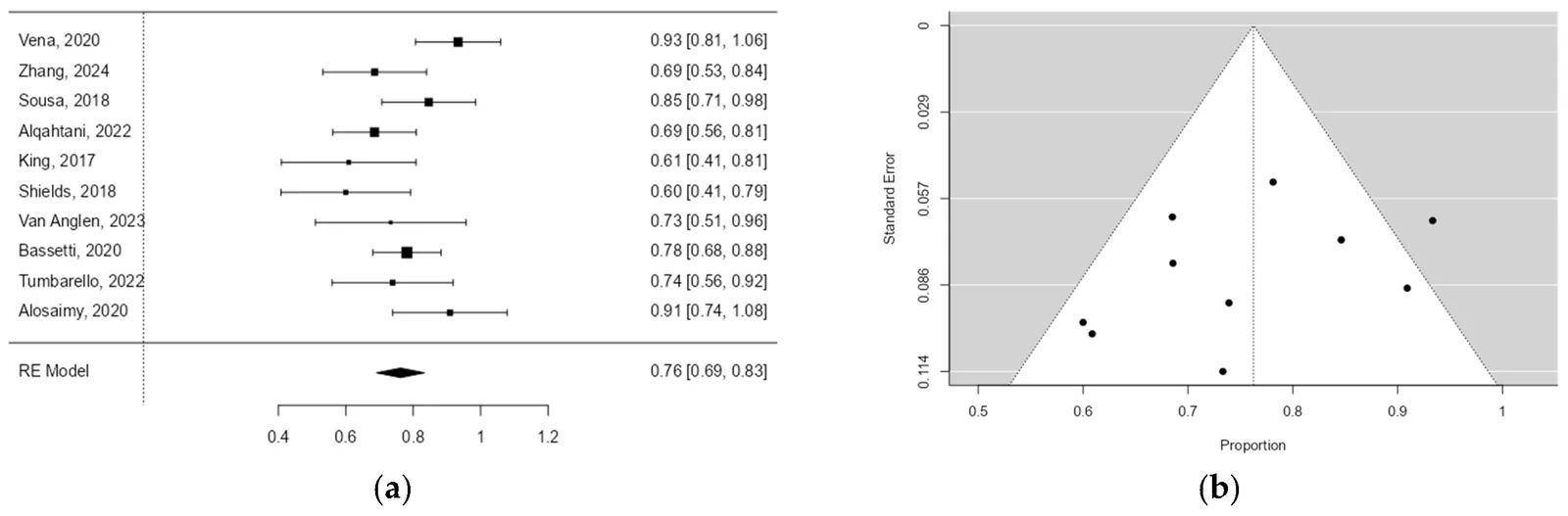
(**a**) Forest plot of pooled clinical cure estimate. (**b**) Funnel plot for assessing publication bias in clinical cure outcome. Symmetry indicates no significant publication bias.

The primary outcomes assessed in this meta-analysis were survival, reported in 90% of the included studies, and clinical cure, reported in 50% of the studies. The pooled survival estimate from 18 studies, derived using a random-effects model, was 73.6% (95% CI: 67.5–79.8%, *p* < 0.001), with moderate heterogeneity (I^2^ = 59.81%) (Figure 2a). Assessment of publication bias via funnel plot asymmetry (Figure 2b), supplemented by regression (*p* = 0.049) and rank correlation tests (*p* = 0.096), suggested a minimal risk of substantial bias. Similarly, the pooled clinical cure estimate from 10 studies was 76.2% (95% CI: 69.2–83.3%, *p* < 0.001), with moderate heterogeneity (I^2^ = 53.38%) (Figure 3a). Regression and rank correlation tests for funnel plot asymmetry (Figure 3b) showed no significant publication bias (*p* = 0.223 and *p* = 0.381, respectively). Since C/A was the most frequently studied antibiotic in this meta-analysis, the results of a subgroup analysis specifically for C/A demonstrated a pooled survival estimate of 73.4% (95% CI: 66.4–80.3%, *p* < 0.001) (Table 2).

The methodological quality of the included studies was assessed using the Methodological Index for Non-Randomized Studies (MINORS) tool and two supplementary criteria: bacteremia population percentage and comprehensiveness of outcome reporting (Table 3). All studies were classified as moderate quality (Category B) based on MINORS scores, reflecting typical limitations of retrospective designs, such as incomplete follow-up data. Regarding bacteremia population percentage, four studies were rated as Category A (>50%), eight as Category B (30–50%), and eight as Category C (<30%). For outcome reporting, six studies were rated as Category A (≥3 outcomes clearly defined), six as Category B (2 outcomes), and eight as Category C (vague or single outcome).

Overall, 8 studies met the criteria for low risk of bias, while 12 studies with smaller bacteremia populations and incomplete outcome reporting were classified as high risk of bias. Subgroup analyses based on study quality showed that studies with a low risk of bias reported a pooled survival estimate of 71.9% (95% CI: 62.2–81.6%, *p* < 0.001) with substantial heterogeneity, whereas studies with a high risk of bias had a slightly higher pooled survival estimate of 75.0% (95% CI: 66.8–83.1%, *p* < 0.001) with moderate heterogeneity (Table 2).

The narrative synthesis focused on a study by Ackley et al. [26], which compared the effectiveness of C/A and M/V. Bacteremia cases accounted for 40% of the study cohort (53/131 patients). Clinical success was achieved in 97.7% of C/A-treated patients and 88.9% of M/V-treated patients. The difference was not statistically significant (*p* = 0.31), indicating comparable effectiveness for both antibiotics in bacteremia caused by CRE. Key differences were noted in treatment dynamics: M/V was more consistently used as monotherapy, with only 15% of patients requiring adjunctive therapy compared with 61% in the C/A group. Resistance development was observed exclusively in the C/A group, raising concerns about its durability as a standalone treatment. While adverse events such as nephrotoxicity were slightly higher in the C/A group, these findings were general to the cohort rather than specific to bacteremia.

## 3. Discussion

The growing burden of MDR bacteremia has intensified the demand for effective, less toxic antibiotic treatments. Among MDROs, CRE and MDRPA represent two of the most significant antimicrobial resistance threats, often necessitating the use of colistin-based regimens, either alone or in combination with aminoglycosides. However, these agents are associated with notable toxicity concerns, highlighting the urgent need for safer and more effective alternatives. The introduction of novel BL/BLIs, including C/A, C/T, and M/V, represents a significant advancement in the management of MDR infections. These agents provide broad-spectrum activity against MDR-GNBs while potentially improving efficacy and reducing toxicity compared with older therapies. However, despite their increasing off-label use for bacteremia, comprehensive data on their real-world effectiveness remain limited. This meta-analysis of 20 observational studies provides valuable insights into the clinical effectiveness of novel BL/BLIs in treating bacteremia, with a pooled survival rate of 73.6% (CI: 67.5–79.8%) and a clinical cure rate of 76.2% (CI: 69.2–83.3%), indicating favorable treatment outcomes. Most studies assessed patients with severe resistant infections and significant comorbidities, such as hematologic malignancies, solid organ transplantation, and COVID-19, further reinforcing the real-world applicability of these findings.

These results align with existing evidence supporting the role of BL/BLIs in treating bacteremia. A meta-analysis by Chen et al. [27], which evaluated the effectiveness of C/A in BSIs, demonstrated a significant reduction in mortality (RR = 0.55, 95% CI: 0.45–0.68, *p* < 0.00001) and improved clinical cure rates (RR = 1.75, 95% CI: 1.57–2.18, *p* < 0.00001) compared with control regimens. Similarly, another meta-analysis by Shields et al. [28], which assessed C/A in nosocomial pneumonia and bacteremia, found that C/A was associated with a significant reduction in mortality (OR = 0.30, 95% CI: 0.19–0.46) and a substantial increase in clinical cure rates (OR = 4.90, 95% CI: 2.60–9.23). These findings are consistent with the sub-analysis of C/A in our meta-analysis, which showed a pooled survival rate of 73.4% (CI: 66.4–80.3%), despite moderate heterogeneity (I^2^ = 63.39%), further reinforcing its role as a key treatment option for MDR bacteremia. C/T has also been studied in the context of bacteremia, as highlighted by Khankhel et al. [29], whose systematic literature review reported favorable cure rates of 33.3–100% for primary, 60–100% for secondary, and 50–91.7% for mixed/unspecified bacteremia, with mortality rates ranging from 0% to 51.6% across groups despite challenges related to sample sizes and study heterogeneity. In contrast, real-world evidence on M/V remains limited, making it difficult to draw definitive conclusions regarding its role in bacteremia. A literature review by Waters & Shorr [30] explored its potential use in CRE bacteremia but emphasized the scarcity of real-world data, cautioning against its routine use given the availability of more well-established alternatives. Similarly, this meta-analysis identified fewer studies on M/V compared with C/A and C/T, underscoring the need for further research to clarify its role in bacteremia. The narrative synthesis of Ackley et al. [26], comparing M/V with C/A, revealed comparable clinical success rates but raised concerns about resistance development with C/A, emphasizing the need for tailored treatment strategies in clinical practice.

Current IDSA guidelines recommend C/A and M/V as preferred treatments for CRE infections and C/T for infections caused by difficult-to-treat (DTR) Pseudomonas aeruginosa, particularly for infections outside the urinary tract [31]. Our findings further support the potential role of these agents in bacteremia, suggesting their consideration as empiric options for patients at high risk of MDR bacteremia to minimize reliance on nephrotoxic alternatives, as timely initiation of appropriate antibiotic therapy remains critical to improving outcomes given the high mortality associated with this condition. At the same time, antimicrobial stewardship programs (ASPs) play a crucial role in ensuring the judicious use of novel BL/BLIs [32,33]. Clinicians should guide antibiotic selection based on local resistance patterns, patient microbiological history, and the overall clinical picture to optimize efficacy while minimizing unnecessary broad-spectrum use. Implementing responsible prescribing practices is essential not only to preserve the effectiveness of these agents but also to mitigate the emergence of further antimicrobial resistance [34,35]. Moreover, continuous surveillance of resistance trends and patient outcomes should be integrated into stewardship initiatives, facilitating the refinement of treatment strategies and promoting the sustainable use of these critical antibiotics.

This meta-analysis offers a comprehensive quantitative synthesis of the real-world use of novel BL/BLIs in bacteremia, providing clinically relevant insights that may inform treatment strategies and optimize patient management. However, several limitations must be acknowledged. The reliance on observational studies introduces potential bias and confounding, restricting definitive causal inferences on the comparative effectiveness of these agents against conventional regimens. Furthermore, the limited representation of M/V relative to C/A and C/T reduces the generalizability of findings across the broader BL/BLIs class. The predominance of studies from high-income countries may further impact applicability to lower-income settings, where antibiotic accessibility, resistance patterns, and healthcare infrastructure differ. Inconsistencies in the reporting of microbiological eradication and recurrence outcomes further impede their integration into quantitative analyses, while the lack of bacteremia-specific safety data constrains a comprehensive evaluation of these agents’ safety profiles in bacteremia.

Despite these limitations, this study has several notable strengths. A systematic and targeted literature search ensured the inclusion of bacteremia-specific data, enabling a comprehensive evaluation of real-world treatment outcomes. The selection of survival and clinical cure as primary endpoints enhances clinical relevance, strengthening the applicability of the findings to clinical practice. Additionally, the use of subgroup analyses and a random-effects model to account for heterogeneity further reinforces the robustness of the conclusions, supporting the clinical utility of these agents in the management of bacteremia.

Future research should prioritize RCTs to validate the effectiveness of these agents and facilitate direct comparisons with alternative therapies. Comparative studies investigating the relative roles of C/T, C/A, and M/V in the management of bacteremia are particularly warranted, especially in immunocompromised and critically ill populations. Furthermore, assessing the pharmacoeconomic impact of these agents would provide essential insights for optimizing their use in resource-limited settings. While this meta-analysis establishes a strong foundation for the clinical application of BL/BLIs, continued research is crucial to further define their role in the treatment of bacteremia and ensure their long-term efficacy in clinical practice.

## 4. Materials and Methods

This systematic review and meta-analysis were conducted in accordance with the Meta-analyses of Observational Studies in Epidemiology (MOOSE) guidelines and adhered to the Preferred Reporting Items for Systematic Reviews and Meta-Analyses (PRISMA) reporting guidelines. A comprehensive search of PubMed and Scopus databases was performed directly through the databases’ interfaces from inception through August 2024, focusing on observational studies evaluating the real-world effectiveness of C/T, C/A, and M/V in treating bacteremia. The search utilized keywords such as ‘Ceftazidime/Avibactam’, ‘Meropenem/Vaborbactam’, ‘Ceftolozane/Tazobactam’, ‘β-lactamase inhibitor’, and ‘bacteremia’, along with their synonyms, employing Boolean operators (AND, OR) to effectively refine results. No date or geographic restrictions were applied; however, the search was restricted to English-language studies due to resource limitations. Reference lists of included studies were also manually screened to identify additional eligible articles.

Observational studies, including retrospective and prospective cohort studies, as well as case series with ≥10 patients, were included if they assessed the use of C/T, C/A, or M/V independently and provided bacteremia-specific survival or clinical cure rates. Studies were excluded if they were in vitro or animal studies, they were microbiological surveillance reports, they had sample sizes < 10, they lacked bacteremia-specific outcomes, or they were RCTs. RCTs were excluded because the objective was to evaluate the real-world effectiveness, not the efficacy, of C/T, C/A, and M/V. Authors S.M. and M.A. evaluated the studies for inclusion. The protocol for this systematic review and meta-analysis was registered in PROSPERO (registration ID: CRD42024610592).

S.M. and M.A. systematically abstracted the data through the collection of study identification details (study name, author, and publication year), study design (type, location, duration, and single/multicenter status), population characteristics (sample size, age, primary and secondary bacteremia proportions, infection sources, and pathogen resistance profiles), interventions (antibiotic regimens, dosing, duration, and concomitant therapies), and outcomes (clinical cure rates and survival, derived from mortality data). The selection and coding process was developed to align with the clinical objectives of assessing treatment effectiveness in bacteremia. Data were meticulously organized using a structured Excel template to ensure consistency and facilitate analysis, with any discrepancies resolved through collaborative consensus.

The methodological quality of the included studies was assessed using the MINORS tool, complemented by two additional criteria: the proportion of bacteremia patients within the study population and the comprehensiveness of clinical outcome reporting, aimed at reducing potential confounding and enhancing study relevance. Each criterion was categorized as Category A (high quality), Category B (moderate quality), or Category C (low quality). MINORS scores ranged from 0 to 16, with studies classified as Category A [15,16], Category B [9,10,11,12,13,14], and Category C (<8). Bacteremia representation was categorized as Category A (>50%), Category B (30–50%), and Category C (<30%). Clinical outcome reporting was classified as Category A (≥3 outcomes), Category B (2 outcomes), and Category C (≤1 outcome).

Studies were classified as low risk of bias if they met Category A across all criteria, had Category A in bacteremia representation and outcome reporting but Category B in MINORS, or had Category B in all three criteria. All other studies, including those with Category C ratings in any criterion, were classified as high risk of bias. Discrepancies in assessments were resolved by consensus.

The primary outcomes for this meta-analysis were survival and clinical cure. Survival was defined as the proportion of patients who survived after treatment with one of the novel antibiotics, based on reported mortality data. Clinical cure was defined as the resolution of signs and symptoms of infection. Studies were eligible for inclusion if they reported at least one of these primary outcomes, allowing for a broader assessment of clinical effectiveness across diverse study designs.

Statistical analyses were conducted using Jamovi with the Proportional Meta-Analysis module, applying a random-effects model to account for variability across studies. Effect sizes for survival and clinical success rates were calculated as raw proportions with 95% confidence intervals (CIs) and independently analyzed. Heterogeneity was assessed using the I^2^ statistic, with values exceeding 50% indicating substantial heterogeneity. Subgroup analyses were performed to examine potential variations in treatment outcomes, specifically assessing the impact of study methodological quality and specific antibiotics on survival outcomes. Publication bias was evaluated through funnel plot analysis, with asymmetry suggesting possible bias.

## 5. Conclusions

This meta-analysis highlights the clinical effectiveness of novel BL/BLIs—C/T, C/A, and M/V—as off-label treatments for bacteremia. Collectively, these agents demonstrated significant therapeutic potential, with C/A being the most extensively studied and exhibiting the most consistent benefits. The findings support the role of these antibiotics in antimicrobial stewardship programs, providing viable alternatives to less effective or more toxic regimens. However, further RCTs and comparative studies are essential to validate these results and refine treatment protocols. These insights contribute to advancing the evidence base for managing bacteremia in diverse clinical settings.

## Figures and Tables

**Table 1:** Demographics of included studies (*n* = 20).

	Study Type	Location	Duration (Months)	Population of Interest	Number of Patients with Bacteremia *n*/*N* (%)	Number of Bacteremia Patients Receiving Novel Antibiotic *n*/*N* (%)	Primary Bacteremia, %	Secondary Bacteremia, %	Source of Infection(s), %	Pathogen Type, %	Resistance Profile, %	Study Drug	Dose	Treatment Duration, Days	Number of Patients Receiving Concomitant Antibiotics, %
[24]	Case Series	Brazil	22	Patients with infections caused by MDR *Enterobacteriales*	12/29 (41.4%)	12/12 (100%)	100%	NR	NR	*KP*: 96.5%, *S. marcescens*: 3.4%	KPC-2: 100%	C/A	2.5 g q8hrs, adjusted based on kidney function	Mean: 12	48.27%
[21]	Retrospective Observational Study	China	29	Patients with CR-GNB infections	31/94 (32.9%)	31/31 (100%)	NR	NR	NR	CRE: 87.09%, CRPA: 12.9%	NR	C/A	2.5 g q8hrs	Median: 8	NR
[12]	Case Series	Italy	25	Patients infected with MDR-GNB other than CRE	15/41 (36.6%)	15/15 (100%)	46.6%	53.3%	RTIs: 25%, CVC: 12.5%, IAIs: 25%, pyelonephritis: 12.5%, heart: 12.5%, mediastinum: 12.5%	Reported only for 1ry bacteremia, *PA*: 71.4%, ESBL-producing *Enterobacterales*: 14.2%, polymicrobial: 14.2%	NR	C/A	2.5 g q8hrs, adjusted based on kidney function	Median: 13	NR
[22]	Retrospective Observational Study	China	54	Kidney transplant recipients with CR-GNB infections	35/81 (43.2%)	35/35 (100%)	NR	NR	NR	NR	NR	C/A	2.5 g q8hrs, adjusted based on kidney function	Median: 14	NR
[18]	Retrospective Observational Study	Spain	21	Patients infected by OXA-48-producing *Enterobacteriaceae*	26/57 (45.6%)	26/26 (100%)	23.1%	76.9%	RTIs: 25%, IAIs: 40%, UTIs: 25%, Other: 10%	NR	OXA-48 CRE	C/A	2.5 g q8hrs, adjusted based on kidney function	Median: 13	NR
[13]	Retrospective Observational study	Italy	21	Patients with KPC-KP infections	104/138 (75%)	104/104 (100%)	NR	NR	NR	*KP*: 100%	KPC: 100%	C/A	2.5 g q8hrs, adjusted based on kidney function	Median: 14	78.8%
[25]	Retrospective Observational Study	KSA	35	Patient infected with CP-CRE	54/211 (25.5%)	54/54 (100%)	NR	NR	NR	*KP*: 94.4%	OXA-48: 72.2%, NDM: 27.7%	C/A	NR	NR	NR
[6]	Retrospective Observational study	USA	30	Patients with CRE bacteremia	137/137 (100%)	32/137 (23.35%)	13.1%	86.8%	IAIs: 37.8%, UTIs: 14.2%, RTIs: 15.1%, GIs: 10.9%, SSTIs: 5.8%, Other: 15.9%	NR	NR	C/A	NR	NR	34.3%
[7]	Retrospective Observational Study	USA	13	Patients infected with CRE	23/60 (38.3%)	23/23 (100%)	NR	NR	NR	NR	NR	C/A	NR	NR	NR
[8]	Retrospective Observational study	USA	24	Patients infected by CRE	20/77 (26%)	20/20 (100%)	100%	0%	NR	NR	NR	C/A	2.5 g q8hrs, adjusted based on kidney function	Median: 14	NR
[14]	Case Series	Italy	6	Patients with BSIs caused by KPC-producing or NDM-producing *Klebsiella pneumoniae* during COVID-19 hospitalization	44/44 (100%)	KPC: C/A: 23/44 (52.2%), NDM: C/A + Aztreonam 21/44 (47.7%)	34%	65.9%	UTIs: 44.8%, CVC: 48.2%, VAP: 6.8%	*KP*: 100%	KPC: 52.3%, NDM: 47.7%	C/A	2.5 g q8hrs	NR	KPC: 70%, NDM: 14%
[9]	Retrospective Observational Study	USA	48	Patients infected by MDR-GNB	10/203 (4.9%)	10/10 (100%)	100%	0%	NR	CRE: 70%, *PA*: 10%	NR	C/A	2.5 g q8hrs, adjusted based on kidney function	Median: 9	NR
[15]	Retrospective Observational Study	Italy	22	High-risk patients with hematological malignancies colonized by KPC-KP who developed BSI	34/34 (100%)	12/34 (35.2%)	NR	NR	NR	*KP*: 100%	CR-KPC: 100%	C/A	2.5 g q8hrs	NR	91.6%
[23]	Retrospective Observational study	India	12	Patients with infections caused by CRO	23/78 (29.4%)	23/23 (100%)	86.9%	13.0%	NR	NR	NR	C/A	2.5 g q8hrs, adjusted based on kidney function	Median: 7	NR
[19]	Retrospective Observational Study	Spain	22	Critically ill patients with infections caused by GNBs	22/68 (32.35%)	22/22 (100%)	NR	NR	NR	NR	NR	C/A	2.5 g q8hrs, adjusted based on kidney function	Median: 10	NR
[10]	Retrospective Observational Study	USA	67	Patients with complicated infections in an outpatient setting	15/126 (11.9%)	15/15 (100%)	20%	80%	BJIs: 41.6%, SSTIs: 16.6%, UTIs: 16.6%, RTIs: 16.6%, IAIs: 8.3%	Reported only for 1ry bacteremia, *PA*: 66.67%, *E. cloacae*: 33.3%	NR	C/T	4.5 g per day	Median: 41	NR
[16]	Retrospective Observational Study	Italy	36	Patients with infections caused by ESBL-producing *Enterobacterales*	64/153 (41.8%)	64/64 (100%)	10.4%	31.3%	NR	NR	NR	C/T	1.5 g q8hrs, adjusted based on kidney function	Median: 14	NR
[20]	Retrospective Observational Study	Spain	45	Critically ill patients with PA infections	15/95 (15.7%)	15/15 (100%)	33.3%	66.6%	UTIs: 10%, RTIs: 80%, SSTIs: 10%	*PA*: 100%	NR	C/T	Standard: 1.5 g q8hrs, high: 3 g q8hrs	Median: 10	NR
[17]	Retrospective Observational Study	Italy	8	Patients with infections caused by KPC-KP	23/37 (62.2%)	23/23 (100%)	NR	NR	NR	*KP*: 100%	KPC: 100%	M/V	4 g q8hrs, adjusted based on kidney function	Median: 13.5	39.1%
[11]	Retrospective Observational Study	USA	20	Patients with serious infections caused by GNBs	11/40 (27.5%)	11/11 (100%)	18.1%	81.8%	NR	NR	NR	M/V	NR	Median: 12	NR

Abbreviations: NR: not reported, MDR: multidrug resistant, KP: *Klebsiella pneumoniae*, C/A: Ceftazidime/Avibactam, CR-GNB: carbapenem-resistant Gram-negative bacteria, CRE: carbapenem-resistant *Enterobacterales*, CRPA: carbapenem-resistant *Pseudomonas aeruginosa*, MDR-GNB: multidrug-resistant Gram-negative bacteria, RTIs: respiratory tract infections, CVC: central venous catheter, IAIs: intra-abdominal infections, UTIs: urinary tract infections, KPC-KP: *Klebsiella pneumoniae* carbapenemase-producing *Klebsiella pneumoniae*, CP-CRE: carbapenemase-producing carbapenem-resistant *Enterobacterales*, GIs: gastrointestinal infections, SSTIs: skin and soft tissue infections, BSIs: bloodstream infections, VAP: ventilator-associated pneumonia, CRO: carbapenem-resistant organisms, GNBs: Gram-negative bacteria, BJIs: bone and joint infections, C/T: Ceftolozane/Tazobactam, PA: *Pseudomonas aeruginosa*, M/V: Meropenem/Vaborbactam.

**Table 2:** Summary of Pooled Rates and Heterogeneity for Survival and Clinical Cure Outcomes with Sub-analyses of High-Quality, Low-Quality, and C/A Studies.

Outcomes	Included Studies	Pooled Rate (CI), %	I^2^ Value, %
Survival	18	73.6 (67.5–79.8)	59.81
Clinical cure	10	76.2 (69.2–83.3)	53.38
Sub-analyses			
Low risk of bias studies	8	71.9 (62.2–81.6)	65.78
High risk of bias studies	10	75.0 (66.8–83.1)	55.91
C/A-only studies	14	73.4 (66.4–80.3)	63.39

**Table 3:** Methodological Quality Assessment of Included Studies.

	Score 1: MINORS Criteria		Score 2: Bacteremia Population Size Criterion		Score 3: Clinical Outcome Reporting		Overall Result
	Score	Interpretation	Score	Interpretation	Score	Interpretation	
[24]	11/16	B	12/29 (41%)	B	2	B	Low risk of bias
[21]	9/16	B	31/94 (33%)	B	1	C	High risk of bias
[12]	9/16	B	15/41 (37%)	B	3	A	Low risk of bias
[22]	9/16	B	35/81 (43%)	B	4	A	Low risk of bias
[18]	11/16	B	26/57 (46%)	B	4	A	Low risk of bias
[13]	9/16	B	104/138 (75%)	A	2	B	Low risk of bias
[25]	9/16	B	54/211 (25%)	C	2	B	High risk of bias
[6]	9/16	B	32/137 (23%)	C	1	C	High risk of bias
[7]	9/16	B	23/60 (38%)	B	3	A	Low risk of bias
[8]	10/16	B	20/77 (26%)	C	1	C	High risk of bias
[14]	9/16	B	44/44 (100%)	A	1	C	High risk of bias
[9]	9/16	B	10/203 (5%)	C	2	B	High risk of bias
[15]	9/16	B	34/34 (100%)	A	1	C	High risk of bias
[23]	10/16	B	23/78 (29%)	C	1	C	High risk of bias
[19]	9/16	B	22/68 (32.35%)	B	1	C	High risk of bias
[10]	9/16	B	15/126 (11.9%)	C	2	B	High risk of bias
[16]	9/16	B	64/153 (42%)	B	2	B	Low risk of bias
[20]	9/16	B	15/95 (16%)	C	1	C	High risk of bias
[17]	9/16	B	23/37 (62%)	A	4	A	Low risk of bias
[11]	9/16	B	11/40 (28%)	C	3	A	High risk of bias

## Data Availability

All data supporting the findings of this study are available within the manuscript. Additional details can be provided upon reasonable request.
